# A Study on the Relationship between Type 2 Diabetes and Taste Function in Patients with Good Glycemic Control

**DOI:** 10.3390/nu12041112

**Published:** 2020-04-16

**Authors:** Sofia Pugnaloni, Sonila Alia, Margherita Mancini, Vito Santoro, Alice Di Paolo, Rosa Anna Rabini, Rosamaria Fiorini, Jacopo Sabbatinelli, Mara Fabri, Laura Mazzanti, Arianna Vignini

**Affiliations:** 1 Department of Clinical Sciences, Section of Biochemistry, Biology and Physics, Università Politecnica delle Marche, Via Tronto 10/A, 60126 Ancona, Italy; s.pugnaloni@pm.univpm.it (S.P.); s.alia@pm.univpm.it (S.A.); alicedippi@gmail.com (A.D.P.); j.sabbatinelli@pm.univpm.it (J.S.); mazzanti.laura@gmail.com (L.M.); 2 Department of Clinical Sciences, School of Specialization in Clinical Nutrition, Università Politecnica delle Marche, Via Tronto 10/A, 60126 Ancona, Italy; mancini.margherita@gmail.com (M.M.); vito.santoro88@gmail.com (V.S.); 3 Diabetology Department, IRCCS INRCA, Via della Montagnola 81, 60127 Ancona, Italy; rosaanna.rabini@sanita.marche.it; 4 Department of Life and Environmental Sciences, Università Politecnica delle Marche, Via Brecce Bianche, 60131 Ancona, Italy; r.fiorini@univpm.it; 5 Department of Experimental and Clinical Medicine, Università Politecnica delle Marche, Via Tronto 10/A, 60126 Ancona, Italy; m.fabri@univpm.it

**Keywords:** diabetes, taste function, gender, food preferences

## Abstract

Type 2 diabetes mellitus (T2DM) has a very high impact on quality of life as it is characterized by disabling complications. There is little evidence about taste alterations in diabetes. Since many individual factors are involved in the onset of diabetes, the purpose of our study is to search a possible link between diabetes and individual taste function. Thirty-two participants with T2DM and 32 volunteers without T2DM (healthy controls) were recruited. Four concentrations of each of the four basic tastes (sweet, sour, salty, bitter), and pure rapeseed oil and water, were applied with cotton pads to the protruded tongue, immediately posterior to its first third, either to the left or right side. The results showed significant differences between groups in the ability to recognize sour, bitter, sweet, and water. Taste scores were lower in subjects with T2DM than in healthy controls, and an age-related decline in taste function was found. The taste function reduction associated with T2DM was not related to gender, disease duration, and glycemic control. In conclusion, it can be hypothesized that a general alteration of taste function can lead patients with type 2 diabetes to search for foods richer in sugars, as in a vicious circle, thus decreasing the likelihood of remission of diabetes mellitus.

## 1. Introduction

Type 2 diabetes mellitus (T2DM) is a multifactorial, complex disease associated with chronic hyperglycemia, resulting from the interplay of genetic, environmental, and epigenetic factors [1]. In T2DM, the combination of insulin resistance and a defective insulin response is associated with disorders in carbohydrate, fat, and protein metabolism [2]. Diabetes represents one of the most challenging health problems of the 21st century: according to a survey by the International Diabetes Federation, there were 463 million adults with diabetes in 2019 worldwide, which corresponds to a striking 9.3% prevalence; this number is expected to increase to 700 million by 2045 [3], with an economic impact on personal medical expenditure and on the healthcare system.

T2DM often presents with mild manifestations, to the point that at the time of diagnosis many patients already present with one or more complications which negatively affect the patient’s quality of life and account for the mortality and morbidity associated with the disease [1,4].

Taste perception and food preferences are shown to be important determinants of dietary practices [5], thus guiding and helping us in the identification and consumption of nutrients. 

For humans, the so-called “basic” tastes are sweet, umami, sour, salty, and bitter [6]; further and more recent research suggests the presence of two additional tastes: fat and water tastes [7,8].

Abnormalities in any or several taste receptors are known to influence intake of specific food components or ingredients related to the taste receptor [9]. An impairment of taste perception, thus possibly shifting towards unhealthy eating behaviors, could have extensive health consequences because it could increase the risk of dysmetabolic diseases such as obesity, diabetes, and metabolic syndrome [10]. Thus, the study of taste physiology and its connections to human health has been receiving increasing attention. 

To date, there are very few reports describing changes in overall taste sensitivity in T2DM. Whether or not environmental influences, such as habitual diet, can alter taste sensitivity, or vice versa, is still unclear. People with diabetes may show impaired taste sensation, mostly for the sweet perception compared to other taste modalities [11,12]. This alteration in sweet taste sensitivity could be responsible for wrong and unbalanced diet choices, thus worsening obesity and visceral fat accumulation, and increasing insulin resistance. As many individual factors are involved in the onset of diabetes, it might be hypothesized that a link exists between diabetes and individual taste sensitivity. 

Thus, the present study was aimed at determining taste function in a population of T2DM subjects, with good metabolic control and without complications, and evaluating possible relations with age, gender, disease duration, and severity. 

## 2. Materials and Methods

### 2.1. Subjects

The present study was performed according to the guidelines of the Declaration of Helsinki as revised in 2001, after the protocol was approved by the Review Board of Università Politecnica delle Marche (Protocol 200446). Written informed consent was obtained from all enrolled subjects after the procedures were fully explained and prior to the anthropometric parameter measurements and taste test execution.

Thirty-two T2DM patients (15 females and 17 males, age 63.3 ± 14.5 years) and 32 non-T2DM healthy volunteers (18 females and 14 males, age 63.9 ± 10.2 years) were enrolled at the Diabetology Unit, IRCCS INRCA, Ancona, from January 2016 to March 2016. Patients were selected according to the following criteria: age between 18 and 65 years, stable and satisfactory glycemic control with glycated hemoglobin (HbA1c) <7% (53 mmol/mol), no sign of neuropathy, retinopathy, or nephropathy, and dietetic therapy only.

Exclusion criteria were: smoking, assumption of any drug affecting taste perception, presence of comorbidity impacting taste (i.e., renal or liver diseases, presence or previous history of cancer, previous head and neck radiation treatment, hypothyroidism, neurodegenerative diseases, depression, acute infections in the previous 2 weeks, respiratory diseases, periodontitis, anosmia, and denture carriers). Furthermore, since it has been reported that more than 250 medications affect smell or taste [13], subjects on any routine medications were also excluded.

The controls were healthy, non-T2DM volunteers, selected in the same period among hospital healthcare professionals and their relatives, and they were matched for sex, age, and body mass index with the patients.

Patients and controls had been following a Mediterranean diet (1800 kcal/die, 22% proteins, 26% lipids, 52% carbohydrates) for at least 6 months before the study. Concerning diabetic patients, adherence to the diet was evaluated by trained nutritionists who took care of giving correct and useful instruction for day-to-day management and food preparation, and meetings were scheduled to verify that patients were correctly following the recommended diet. 

No significant change in weight was observed in participants in the last three months before the study.

Participants were asked to avoid eating and drinking anything except water and not to brush their teeth for one hour prior to testing. Body weight, height, and blood pressure were also measured at enrollment.

### 2.2. Taste Test

The taste test, performed at the Diabetology Unit, was based on filter paper strips [14], modified as described in our previous work [15]: cotton pads, soaked with four substances (sodium chloride, citric acid, sucrose, and quinine hydrochloride) were applied to the protruded tongue, immediately posterior to its first third, either to the left or right side, in order to study lateralization too; each basic taste quality (salty, sour, sweet, and bitter) was presented at 4 different concentrations (Table 1); in addition, pure rapeseed oil and water were administered to evoke fat and neutral taste, respectively. Rapeseed oil is a neutral oil which has a pale yellow color and is almost odorless; rapeseed oil was chosen instead of olive oil since the latter has a specific texture and increased volatility in the oral cavity, making it easily recognizable.

Distilled water was used as a solvent, and taste solutions were freshly prepared on the morning of each testing session. 

Since gustatory stimulation also causes the activation of another sensory system (e.g., touch receptors), the test was performed so as to minimize the activation of other receptors: the stimuli were applied to the left or right side of the protruded tongue, just posterior to the anterior third, by a cotton swab soaked in the different tastant solutions. Subjects were required to wash their mouth with deionized water between samples to avoid carryover effects. 

Administration was randomized for the four concentrations and the side of presentation was alternated: 36 cotton pads (18 for the left side and 18 for the right side) were used. The enrolled subjects had to identify the taste by choosing from a list that included eight descriptions: sweet, salty, bitter, sour, water, fat, nothing, I do not know (forced multiple choice). The test took about 20 min.

### 2.3. Statistical Analysis

Statistical analysis was conducted using IBM SPSS Statistics version 23.0 (IBM Co., Armonk, NY, USA). A *p*-value < 0.05 was deemed as significant. The data used in this study were answers repeatedly collected from the same subject for the various types of stimuli. Results are expressed as means ± SD. We evaluated the number of right answers according to the stimulus and as a function of other physiological and pathological parameters previously recorded (age, sex, arterial pressure, diabetes mellitus). Prior to statistical analysis, the distribution of the data was tested for normality using the Shapiro–Wilk test. The distribution of the correct answer rates was normal in each group.

The overall relationship among taste perception, disease duration, age, and type of stimulation was analyzed using generalized estimating equations (GEE). GEE represent an extension of the generalized linear model by providing support for correlated data, such as repeated measures. In GEE, between-subject and within-subject correlations are taken into account, resulting in a single regression coefficient. The repeated measurements included individuals as subject variables and type of stimulation, substance concentrations, and side of stimulation as intra-subject variables. Answers were used as dependent binary variables, assigning value = 0 to incorrect answers and value = 1 to correct answers. Gender, age, disease duration, and characteristics of stimulation were used as independent variables. In GEE, disease duration was treated as a continuous variable. For a better presentation of the results, stimuli concentrations were log-transformed, while age was computed as a 5-unit increase. Adjusted odds ratios and standard deviations were determined with 95% confidence intervals.

One-way analysis of covariance (ANCOVA) was performed to compare taste perception between groups after adjusting for age and gender. The combined effects of T2DM, gender, and type of stimulation on taste function were evaluated using two-way analysis of variance (ANOVA). Linear regression with Pearson correlation was used to assess the correlation between correct answer rates and other continuous variables. Effect sizes were indicated using Cohen’s d and partial eta-squared.

## 3. Results

### 3.1. Study Population

Of a total of 55 T2DM patients recruited, only 32 met the selection criteria and were enrolled for the study. The characteristics of the T2DM patients and 32 control subjects are described in Table 2. The two groups were similar for age, gender distribution, and body mass index (BMI), and differed only for fasting glucose values, as expected. Individual data for each type of stimulus are reported in Supplementary Table S1.

### 3.2. Multivariate Analysis

Generalized estimating equations (GEE) were used for multivariate analysis. Table 3 shows the results of GEE. The results confirm that overall taste discrimination is negatively affected by diabetes and age, with no difference between genders. Specifically, diabetic patients show lower taste discrimination (odds ratio 0.54; CI 0.34–0.85), while every increase of 5 years of age decreases the odds of success by 6.5%. In addition, GEE confirm the negative relation between decreasing stimuli concentration and correct answers rate.

### 3.3. Univariate Analysis

To gain further insights into the differences in taste function between control subjects and T2DM patients, a two-way ANOVA was conducted for each taste (salty, acid, bitter, sweet, fat, water). The rate of correct answers was used as a dependent variable. There was a statistically significant interaction between group and type of stimulation on taste function (F [5,2286] = 3.331, *p* = 0.005, partial η^2^ = 0.007). Results of the simple main effects analysis with multiple pairwise comparisons, summarized in Table 4, showed significant differences between groups in the ability to recognize all stimuli except fat. Since the presence of a “nothing” option among the possible responses could negatively impact on water recognition, we evaluated the proportion of “nothing” over incorrect answers and found no significant difference between the two groups (χ^2^ test; control, 6/31; T2DM, 9/56; *p* = 0.698). Similarly, water discrimination remained significantly lower in T2DM subjects when considering the “nothing” answers as correct (58.6% ± 44.5% vs. 26.6% ± 44.0%, respectively; *p* = 0.006). The overall taste discrimination was lower in T2DM patients (51.8% ± 17.5% vs. 70.8% ± 14.6%, *p* < 0.001, Cohen’s d = 1.18 [95% CI 0.63–1.69]). Differences in overall taste perception remained significant after adjustment for multiple comparisons.

A one-way ANCOVA was performed to determine the effect size of diabetes after adjusting for age and gender. The partial eta-squared indicates a medium effect size of diabetes on overall taste function (F [1,60] = 4.626, *p* = 0.036, partial η^2^ = 0.072).

We then explored the combined effects of gender and group (control/T2DM) on taste function by performing multiple two-way ANOVA tests. As expected, there was no statistically significant interaction between gender and group on overall taste function (F [1,60] = 0.238, *p* = 0.627, partial η^2^ = 0.004), as well as on each type of stimulation (data not shown). The subsequent analysis of simple main effects confirmed a significant effect of T2DM on taste function (F [1,60] = 24.540, *p* < 0.001, partial η^2^ = 0.290), while no main effect of gender was reported (F [1,60] = 0.550, *p* = 0.461, partial η^2^ = 0.009).

A positive correlation between stimulus concentration and the proportion of correct answers was observed for sour, bitter, and sweet stimulations (Table 5), whereas no correlation was found regarding side of stimulation (data not shown).

In addition, a Pearson product–moment correlation revealed a significant age-related decrease in the ability to correctly recognize the various typologies of tastes (F [1,62] = 20.777; r = −0.501; *p* < 0.001). Finally, no significant relationship was found in T2DM subjects between correct answers rate and glycemic control, evaluated as HbA1c levels (F [1,30] = 1.598; r = −0.225; *p* = 0.216).

## 4. Discussion

In the present study, we evaluated the perception of basic tastes in 32 subjects with T2DM compared to 32 healthy volunteers, also taking into account gender, age, tastant concentration, duration of the disease, and glycemic control.

It has been largely demonstrated that the sense of taste is an important tool in the regulation of nutrient ingestion, in digestive process control, and in the release of neuroendocrine hormones for hunger and satiety. Many studies have focused on changes in taste sensitivity in both physiological and pathological situations [16,17]. 

Our results are in accordance with those showing that taste function in humans decreases with age [18]: in the present study, a reduction in taste recognition ability was described with increasing age. Such results remained significant even after multivariate analysis. This decreasing taste acuity might be due to different causes among which a reduction in the number and density of taste buds of the tongue and laryngeal surface of the epiglottis have been reported [19]. 

The interactions between taste and diabetes are very complex issues because eating behavior can be influenced by different aspects (genetic, environmental, behavioral, sensory), in which diabetes mellitus finds some of its possible etiologies. Nowadays, not many works are focused on the relationship between the perception of the different taste modes and T2DM.

In accordance with our results, a general decrease in taste function has been demonstrated in patients with diabetes [20], particularly concerning the sweet taste [21]. An increase in taste threshold has been shown to be associated with hyperglycemia [22]. A significant correlation between taste thresholds and plasma glucose concentration has been described in a previous study, suggesting that patients with T2DM are almost insensitive to the sweet taste response [22]. Our results do not show any relationship between correct answers rate and HbA1c levels; more interestingly, a negative correlation was found between the correct answers rate and disease duration. 

Individuals less sensitive to sweetness could be at risk of long-term health outcomes, such as diabetes, as they need to introduce more sugar compared to more sensitive people, to obtain the same taste sensation [17]. 

Although there is no conclusive evidence suggesting that the decrease in sweet taste function in T2DM patients results from an alteration in glucose homeostasis, or vice versa, the reduced sensitivity to sweet taste might explain the development of a vicious circle leading to a deterioration of glycemic control. Different nutritional surveys have described the presence of a significant prevalence of sweet (habitually soft and palatable) foods in the diet of elderly people [19].

In the present study, a general decrease in taste recognition ability has been found, except for fat stimuli, which were less perceived by both the control group and the T2DM group, in line with the lack of validation for this type of stimulation. Notably, the ability to recognize water stimuli was significantly reduced in subjects with T2DM. To our knowledge, this is the first report of such an alteration in diabetic patients. In recent studies on Drosophila [23,24], humans, and rats [25], a strict interplay has been shown between sugar and water intake regulation, and they seem to be opposite to each other. Petsakou and Perrimon suggest that “the neuronal networks that integrate internal nutrient abundance signals and couple them to homeostatic behaviors remain largely unexplored, and yet disruption of this machinery could potentially contribute to the onset of homeostatic disorders like obesity-linked diabetes” [23]. In addition, ghrelin, a key internal signal for hunger in mammals, is sufficient not only to promote feeding, but also to inhibit water consumption in rats [26].

While we previously identified some gender-related differences in taste function among healthy and diseased individuals [15,27], in the present study, we did not show any significant effect of gender on the differences in taste function between healthy subjects and T2DM patients.

Moreover, in agreement with Gondivkar et al. [21], no correlation regarding side of stimulation was found; thus, taste function was equivalent on left and right sides of the tongue.

Some limitations, however, apply to our findings, that need to be addressed. First of all, the sample size was relatively small, even if it was well-characterized. Second, no validated questionnaire on food consumption was administered. Moreover, we modified the original protocol by Landis et al. [14] by using cotton swabs and by introducing water and fat stimuli. The presence of “nothing” among the possible answers to the stimulation could have produced significant alterations in the scores of water recognition. However, we did not observe any difference in the distribution of “nothing” answers between control and T2DM participants. Regarding fat stimulation, the relatively low recognition scores obtained by participants in both groups support the need for further validation of this non-classical type of stimulation.

To rule out any potential bias related to different glycemic control, to the presence of T2DM complications, and to the different therapies, only T2DM patients with stable and satisfactory glycemic control, without complications, and under diet therapy only were enrolled. Indeed, T2DM patients with poor glycemic control are more at risk of developing complications. Among them, periodontitis and sensory neuropathy are implicated in possible taste disturbances. Periodontitis and oral cavity lesions are responsible for pain related to chewing and swallowing [28]. Moreover, cranial diabetic neuropathy alters nervous transmission with possible consequential changes in taste perception. In addition, the involvement of the autonomic nervous system results in salivary imbalance with effects on chewing and swallowing food [29]. The T2DM subjects selected for the present study were not affected by neuropathy, retinopathy, or nephropathy; thus, the hypothesis that the observed alterations in taste perception are caused by a neurological defect can be excluded.

## 5. Conclusions

In conclusion, on the basis of the present data, it can be hypothesized that a general decrease of taste function can lead T2DM patients to search for foods richer in sugars, as in a vicious circle, thus decreasing the likelihood of remission of diabetes mellitus.

Therefore, present knowledge regarding taste recognition may be used in dietary counseling in order to formulate a diet that takes into account individual sensory characteristics, and to obtain a feeling of fulfillment and satisfaction without providing a high level of simple glucose with consequent health risks. 

Further investigations should be performed on the response of patients to dietary and pharmacological treatment based on the results of the taste test.

## Figures and Tables

**Table 1:** Concentrations of taste stimuli.

Stimulus	Substance	Concentration
Sweet	Sucrose	0.05 g/mL 0.1 g/mL 0.2 g/mL 0.4 g/mL
Salty	Sodium chloride	0.016 g/mL 0.04 g/mL 0.1 g/mL 0.25 g/mL
Bitter	Quinine hydrochloride	0.0004 g/mL 0.0009 g/mL 0.0024 g/mL 0.006 g/mL
Sour	Citric acid	0.05 g/mL 0.09 g/mL 0.165 g/mL 0.3 g/mL
Fat	Rapeseed oil	Pure
Neutral	Deionized water	Pure

**Table 2:** Clinical and demographic characteristics of enrolled subjects.

	Control Subjects (*n* = 32)	T2DM Patients (*n* = 32)	*p*-Value
Age (years)	63.9 ± 10.2	63.3 ± 14.5	NS
Gender (M/F)	14/18	17/15	NS
Diabetes duration (years)	-	10.8 ± 8.7	-
BMI (kg/m^2^)	27.1 ± 4.0	27.6 ± 4.6	NS
Fasting glucose (mmol/L)	5.3 ± 0.9	7.1 ± 0.4	*p* < 0.05
Glycated hemoglobin (mmol/mol)	-	52 ± 0.7	-
Serum creatinine (μmol/L)	89.8 ± 8.8	99.5 ± 8.4	NS
Total cholesterol (mmol/L)	4.68 ± 0.34	4.81 ± 0.27	NS
LDL cholesterol (mmol/L)	2.49 ± 0.17	2.69 ± 0.15	NS
HDL cholesterol (mmol/L)	1.28 ± 0.14	1.26 ± 0.11	NS
Triglycerides (mmol/L)	2.09 ± 0.16	2.13 ± 0.12	NS

T2DM, type 2 diabetes mellitus; BMI, body mass index. NS, not significant.

**Table 3:** Generalized estimated equations model.

Parameter	B	SE	*p*	OR (CI 95%)
Intercept	−0.108	0.470	-	-
Group				
T2DM	−0.622	0.237	0.009	0.54 (0.34–0.85)
Control	reference			1
Gender				
Male	−0.240	0.180	0.184	0.79 (0.55–1.12)
Female	reference			1
Type of stimulation				
Salty	2.214	0.333	<0.001	9.16 (4.76–17.60)
Sour	2.415	0.301	<0.001	11.20 (6.20–20.21)
Bitter	3.553	0.456	<0.001	34.90 (14.28–85.30)
Sweet	2.205	0.325	<0.001	9.07 (4.80–17.16)
Fat	−0.619	0.317	0.051	0.54 (0.29–1.00)
Water	reference			1
Side of stimulation				
Right	0.106	0.095	0.262	0.90 (0.75–1.08)
Left	reference			1
Log_10_ concentration	−0.746	0.132	<0.001	0.47 (0.37–0.61)
Disease duration (years)	−0.017	0.011	0.147	0.98 (0.96–1.01)
Age (5-year increase)	−0.067	0.017	0.029	0.94 (0.88–0.99)

B, beta coefficient; SE, standard error; OR, odds ratio.

**Table 4:** Comparison of taste function between control subjects and T2DM patients.

Type of Stimulation	Correct Answers (%)		Adjusted *p*-Value
	Controls	T2DM	
Salty	68.3 ± 28.6	55.1 ± 30.3	0.001
Sour	81.6 ± 24.6	59.4 ± 28.9	<0.001
Bitter	71.9 ± 29.4	57.0 ± 25.4	<0.001
Sweet	80.5 ± 23.5	53.9 ± 30.7	<0.001
Fat	22.4 ± 20.6	18.8 ± 10.5	0.659
Water	48.3 ± 43.3	12.5 ± 11.1	<0.001
Overall	70.8 ± 14.6	51.8 ± 17.5	<0.001

Data are mean ± SD of correct answers for each stimulus. *p*-values for pairwise comparisons of each simple main effect with Bonferroni–Dunn correction for multiple comparisons.

**Table 5:** Evaluation of taste function according to concentration of the tastant solutions.

Type of Stimulation	df	χ^2^	*p*
Salty	3	4.847	0.183
Sour	3	9.457	0.024
Bitter	3	20.467	< 0.001
Sweet	3	9.621	0.022

The results of multiple χ^2^ tests are reported. df, degrees of freedom.

## Supplementary Materials

The following are available online at https://www.mdpi.com/2072-6643/12/4/1112/s1, Table S1: Individual answers for each stimulus.
