# Supplementary material for: A Study on the Relationship between Type 2 Diabetes and Taste Function in Patients with Good Glycemic Control
Source: Nutrients. 2020 Apr 16;12(4):1112. doi: 10.3390/nu12041112 (PMC7230282; doi:10.3390/nu12041112)
Supplement: Supplementary file 1 [file nutrients-12-01112-s001.pdf]

Supplementary Table 1. Individual answers for each stimulus. 1 for correct answers, 0 for incorrect/"nothing"/"doesn't know" answers. CTRL, control subjects; DM, diabetic patients.

	Stimulus	SALTY	SALTY	SALTY	SALTY	SALTY	SALTY	SALTY	SALTY	SOUR	SOUR	SOUR	SOUR	SOUR	SOUR	SOUR	SOUR	BITTER	BITTER	BITTER	BITTER	BITTER	BITTER	BITTER	BITTER	SWEET	SWEET	SWEET	SWEET	SWEET	SWEET	SWEET	SWEET	FAT	FAT	WATER	WATER
	Concentration (g/mL)	0.25	0.25	0.1	0.1	0.04	0.04	0.016	0.016	0.3	0.3	0.165	0.165	0.09	0.09	0.05	0.05	0.006	0.006	0.0024	0.0024	0.0009	0.0009	0.0004	0.0004	0.4	0.4	0.2	0.2	0.1	0.1	0.05	0.05	-	-	-	-
	Side of stimulation	right	left	right	left	right	left	right	left	right	left	right	left	right	left	right	left	right	left	right	left	right	left	right	left	right	left	right	left	right	left	right	left	right	left	right	left
Group	SUBJECT_ID																																				
CTRL	1	1	1	1	1	1	1	1	1	1	1	1	1	1	1	1	1	1	1	1	1	1	0	1	1	1	1	1	1	1	1	1	0	1	1	1	1
CTRL	2	1	1	1	0	1	1	1	1	1	1	1	1	1	1	1	1	1	1	1	1	1	1	1	1	1	1	1	1	1	1	1	1	0	0	1	0
CTRL	3	1	1	1	1	1	1	1	0	1	1	1	1	1	1	1	1	1	1	1	1	1	1	1	1	1	1	1	1	1	1	1	1	0	0	0	0
CTRL	4	0	0	0	0	1	1	0	0	1	1	1	1	1	1	0	0	1	1	0	0	0	0	0	0	1	1	1	1	1	1	1	1	1	0	0	1
CTRL	5	1	1	1	1	1	1	1	1	1	1	1	1	1	1	1	1	0	1	0	0	0	0	0	0	1	1	0	1	0	1	0	0	1	0	1	1
CTRL	6	1	0	0	1	0	1	0	0	0	0	1	0	1	0	0	0	0	1	1	0	1	0	1	0	0	1	1	1	0	1	0	1	0	0	1	1
CTRL	7	1	1	0	1	1	0	1	1	1	1	1	1	1	0	1	1	1	0	1	1	1	1	1	0	1	1	0	0	1	1	0	1	0	0	1	1
CTRL	8	1	0	1	1	0	0	1	1	1	1	1	1	1	1	1	1	1	1	1	1	0	1	0	1	1	1	1	1	1	1	1	1	0	0	0	0
CTRL	9	1	1	1	1	1	1	1	1	1	1	1	1	0	1	0	1	1	1	1	1	1	1	1	1	1	1	1	1	1	1	1	1	0	0	0	0
CTRL	10	0	0	0	1	1	1	1	0	1	1	1	1	1	0	1	1	1	1	1	1	1	1	1	0	1	1	0	0	1	1	1	1	0	0	1	1
CTRL	11	0	0	1	1	1	1	1	0	0	0	0	0	0	0	0	0	1	1	1	1	1	1	1	1	1	1	1	1	1	1	1	1	0	0	0	0
CTRL	12	1	1	1	1	1	1	1	1	1	1	1	1	1	1	1	1	1	1	1	1	1	1	1	1	1	1	1	0	1	1	1	1	1	1	1	1
CTRL	13	1	1	1	1	1	1	1	1	1	1	1	1	1	1	1	1	1	1	1	1	1	1	1	1	1	1	1	1	1	1	1	1	1	0	0	0
CTRL	14	1	1	1	1	0	0	0	0	1	1	1	0	1	1	0	0	1	1	0	0	0	0	0	0	1	1	1	1	1	1	0	0	1	0	1	0
CTRL	15	1	1	1	0	1	0	1	0	1	1	1	0	0	0	0	0	1	1	1	1	1	1	1	0	1	0	1	1	0	1	1	1	0	0	0	0
CTRL	16	1	1	0	0	0	0	0	0	1	1	1	1	1	1	1	1	1	1	1	1	0	1	1	1	1	1	1	1	1	1	1	1	1	0	1	0
CTRL	17	0	1	1	1	0	1	0	0	1	1	1	1	1	1	1	1	0	0	1	0	1	1	0	0	1	1	1	1	1	1	1	1	0	0	0	1
CTRL	18	1	1	1	1	1	1	1	1	1	1	0	0	1	1	0	0	1	1	1	1	1	1	1	0	1	0	1	0	1	1	1	1	0	0	1	1
CTRL	19	1	1	0	0	1	0	1	0	1	1	1	1	1	1	1	0	1	0	1	0	0	0	0	0	1	0	1	0	1	0	0	0	0	0	0	0
CTRL	20	1	1	0	1	1	1	1	0	1	1	1	1	1	1	0	1	1	1	1	1	1	1	1	1	1	1	1	1	1	1	1	1	0	1	0	0
CTRL	21	1	1	1	0	0	1	1	0	1	1	0	1	1	1	0	1	1	1	1	1	1	1	0	0	1	1	1	1	1	1	1	1	0	0	1	0
CTRL	22	0	0	0	1	0	1	0	0	1	1	1	1	1	1	1	1	1	1	1	1	1	1	0	1	0	1	0	0	1	0	0	0	1	0	0	1
CTRL	23	1	1	1	1	0	1	1	1	1	1	1	1	1	1	1	0	0	0	0	0	0	1	0	0	1	1	1	1	1	1	1	0	0	0	1	0
CTRL	24	0	0	0	0	0	0	0	0	1	0	1	0	1	1	1	0	1	1	1	1	1	1	1	1	0	0	1	0	1	0	0	0	0	0	0	0
CTRL	25	0	0	1	1	1	1	1	1	1	1	1	1	1	1	1	1	1	1	1	1	1	1	0	1	0	1	1	1	1	1	0	1	0	1	0	0
CTRL	26	1	1	1	1	1	1	1	1	1	1	1	1	0	1	0	0	0	1	0	1	0	0	1	0	1	1	1	1	1	1	1	0	1	0	1	1
CTRL	27	0	0	0	1	0	1	0	1	1	1	1	1	1	1	1	1	1	1	1	1	1	1	1	1	1	1	1	1	1	1	1	1	0	0	1	1
CTRL	28	1	0	1	0	1	0	0	0	1	1	1	1	1	1	1	1	1	1	1	1	0	1	1	0	1	1	1	1	1	1	1	1	0	1	1	1
CTRL	29	1	1	1	1	1	1	1	1	1	1	1	1	1	1	0	1	1	1	1	1	1	1	1	0	0	0	1	0	1	1	1	0	0	0	1	0
CTRL	30	1	1	1	1	1	1	1	1	1	1	1	1	1	0	1	1	1	1	0	1	0	1	1	1	0	0	1	1	0	1	1	0	0	0	0	1
CTRL	31	1	1	1	1	1	1	0	0	1	1	1	1	1	1	1	0	0	1	1	0	1	1	0	1	1	1	1	1	1	1	1	0	1	0	0	1
CTRL	32	1	1	1	1	1	1	1	0	1	1	1	1	1	0	1	1	1	1	0	0	1	0	0	0	1	1	1	1	1	1	1	1	0	0	0	0
DM	33	0	0	0	0	1	1	1	1	1	1	0	1	0	0	0	0	0	1	0	1	0	0	0	0	0	1	0	1	0	1	0	0	0	0	0	0
DM	34	0	0	0	1	1	0	1	1	1	1	0	1	0	0	0	1	0	0	1	1	1	0	0	0	0	0	0	0	0	0	0	0	0	0	1	1
DM	35	1	1	1	1	1	1	1	1	0	0	1	1	0	0	0	1	1	0	0	1	0	0	0	0	1	0	0	1	0	0	0	0	0	0	0	0
DM	36	1	1	1	1	1	1	1	1	1	1	0	1	1	1	1	1	1	1	0	1	0	0	1	1	1	1	1	1	1	1	0	1	1	0	0	0
DM	37	0	0	0	0	0	1	0	0	1	1	1	1	1	1	0	0	1	1	0	1	1	0	0	0	1	1	0	1	0	0	0	0	0	0	0	0
DM	38	1	1	0	1	1	1	1	1	0	0	1	1	1	1	1	1	1	1	1	1	0	1	1	1	1	1	1	1	1	1	1	0	1	0	0	0
DM	39	1	1	0	0	0	1	0	0	1	1	1	0	0	0	0	0	1	0	0	1	0	0	0	0	0	0	0	0	0	0	0	0	0	0	0	0
DM	40	0	1	1	0	0	0	0	0	1	0	0	1	1	0	0	1	1	0	1	1	0	1	0	1	1	0	0	1	1	0	0	1	1	0	0	0
DM	41	1	0	0	0	1	0	0	0	1	1	1	1	1	1	1	1	1	1	1	1	0	1	0	1	1	1	0	0	1	1	0	1	0	0	0	0
DM	42	0	0	0	1	0	1	1	1	1	1	1	1	1	1	1	1	1	0	0	1	0	1	0	0	1	1	0	1	1	1	1	1	0	0	0	0
DM	43	1	0	1	1	0	1	0	1	1	1	1	1	1	1	1	1	0	1	1	1	0	1	0	1	0	1	1	1	1	1	0	1	1	0	1	0
DM	44	1	1	1	1	0	0	1	1	1	1	1	1	1	1	0	1	1	1	0	0	1	0	0	1	1	0	0	1	1	0	0	0	0	0	0	0
DM	45	0	0	0	1	0	1	0	1	0	0	0	0	0	0	0	0	0	0	0	0	0	0	0	0	1	1	1	1	1	1	0	1	0	0	0	0
DM	46	0	1	0	1	0	1	1	1	0	0	1	0	0	0	0	0	1	1	1	1	1	1	0	1	0	1	0	1	0	1	1	1	1	1	0	0
DM	47	1	1	1	0	1	1	1	1	1	1	0	0	0	0	0	0	0	1	1	1	0	0	0	1	1	1	1	0	1	0	0	0	0	0	0	0
DM	48	0	0	0	1	0	1	0	1	1	1	1	1	1	1	1	1	1	1	1	1	1	1	1	1	1	0	1	1	0	1	0	1	0	1	0	1
DM	49	0	0	0	0	0	0	0	1	0	1	1	1	1	0	0	1	0	0	0	0	1	0	0	0	0	1	0	0	0	0	0	1	0	0	0	0
DM	50	0	0	1	1	0	1	1	1	0	1	0	0	0	1	0	1	0	1	1	1	0	1	0	0	1	1	0	1	1	1	1	0	0	0	0	0
DM	51	0	0	0	1	0	0	1	0	0	0	0	0	0	1	0	0	1	0	1	0	0	1	0	0	1	1	0	0	0	0	0	0	0	0	0	0
DM	52	1	1	1	1	1	1	1	1	0	1	0	1	1	0	0	0	1	1	1	1	1	1	1	1	1	1	1	1	1	1	1	0	0	0	0	0
DM	53	1	1	1	1	1	0	1	0	0	1	0	0	0	1	0	1	1	0	0	0	1	1	1	0	1	1	1	1	1	1	1	0	0	0	0	0
DM	54	0	0	1	0	1	1	1	1	1	1	0	1	1	1	1	0	1	1	1	1	1	0	0	0	1	1	1	1	1	1	1	0	0	0	0	0
DM	55	1	1	1	1	1	1	1	1	0	1	0	0	0	0	0	1	0	1	1	1	0	0	1	1	0	1	1	1	1	1	1	1	0	0	0	0
DM	56	0	0	0	0	1	1	0	1	0	1	1	1	1	1	1	1	1	1	1	1	1	0	1	1	1	1	1	1	1	1	1	0	0	1	0	0
DM	57	1	1	1	1	1	1	1	1	1	1	1	1	1	0	0	1	1	1	1	1	1	1	1	1	0	1	1	1	1	0	1	1	0	0	0	0
DM	58	0	0	0	0	0	0	0	0	1	1	0	0	1	0	0	0	1	1	1	0	1	1	0	0	1	1	0	1	0	0	0	0	1	0	0	0
DM	59	1	1	1	1	0	0	0	1	1	1	1	1	0	1	1	0	1	1	1	1	0	0	0	1	1	0	1	0	0	0	0	0	0	0	0	0
DM	60	1	0	0	1	0	1	1	0	1	1	1	1	1	1	1	0	0	0	1	1	1	1	0	1	1	0	1	1	1	1	1	0	1	0	1	1
DM	61	0	0	0	1	1	1	0	0	1	1	0	1	1	1	1	1	1	1	0	0	0	1	0	0	0	0	0	0	0	0	0	1	0	0	0	0
DM	62	0	1	0	0	0	1	1	1	1	1	0	0	1	1	0	0	1	1	1	1	0	1	0	0	1	1	0	1	1	0	1	1	1	1	0	0
DM	63	0	0	0	0	0	0	0	0	1	1	1	1	1	0	0	0	1	1	0	1	1	0	0	0	0	0	0	1	0	0	0	0	0	0	0	0
DM	64	0	1	1	1	1	1	1	1	0	1	1	1	1	1	1	0	1	1	1	1	1	1	0	1	0	0	0	0	0	0	0	0	0	0	1	1
